# Night-Time Non-dipping Blood Pressure and Heart Rate: An Association With the Risk of Silent Small Vessel Disease in Patients Presenting With Acute Ischemic Stroke

**DOI:** 10.3389/fneur.2021.719311

**Published:** 2021-11-16

**Authors:** Naveed Akhtar, Salman Al-Jerdi, Saadat Kamran, Rajvir Singh, Blessy Babu, Mohamed S. Abdelmoneim, Deborah Morgan, Sujatha Joseph, Reny Francis, Ashfaq Shuaib

**Affiliations:** ^1^The Neuroscience Institute, Hamad Medical Corporation, Doha, Qatar; ^2^Weill Cornell Medicine, Doha, Qatar; ^3^Neurology Division, Department of Medicine, University of Alberta, Edmonton, AB, Canada

**Keywords:** ischemic stroke, night-time non-dipping, outcome, stroke types, stroke severity, small vessel disease

## Abstract

**Background and Purpose:** Nocturnal non-dipping blood pressure and heart rate are associated with an increased risk of cardiovascular disease. The effects of such variance on cerebrovascular disease have not been well studied.

**Methods:** The 24-h ambulatory blood pressure (ABPM) and heart rate were monitored with B-pro in patients with acute stroke within the initial week of hospital admission. The risk factor profiles, clinical presentation, imaging, and short-term prognosis were compared in nocturnal dippers and non-dippers (more than 10% nocturnal decrease) of blood pressure and heart rate.

**Results:** We enrolled 234 patients in whom ABPM and MRI data were available. Heart rate data were available in 180 patients. Lacunar sub-cortical stroke was the most common acute lesion (58.9%), while hypertension (74%) and diabetes (41.5%) were the most common associated risk factors. ABPM revealed non-dipping in 69% of patients. On univariate analysis, Small Vessel Disease (SVD) was significantly more frequent in non-dippers vs. dippers (BP: 56.8 vs. 40.3% *p* = 0.02; heart rate: 57.9 vs. 40.7% *p* = 0.03). Silent strokes were also more frequent in non-dippers vs. dippers (BP: 40.7 vs. 26.4% *p* = 0.35; heart rate: 44.6 vs. 25.4% *p* = 0.01). Multivariate analysis revealed SVD to be significantly related to age, hypertension, blood pressure non-dipping, and severity of symptoms at index event.

**Conclusions:** The presence of nocturnal non-dipping of blood pressure and heart rate are associated with an increased risk of silent stroke and SVD. Increased use of ABPM may allow for improved diagnosis of non-dippers.

## Introduction

In 1988 O'Brian et al. reported in a study of 102 hypertensive patients that non-dippers (17% of enrolled subjects) were at a higher risk of stroke during follow-up (1). Subsequent studies have shown a strong association between of the incidence of atherosclerosis, coronary artery disease (CAD), and stroke in non-dippers (2–4). Although the exact pathophysiology of non-dipping remains unclear, abnormalities in vascular tone, autonomic dysfunction, and neurohormonal factors all increase the risk of non-dipping (2). Common disorders associated with non-dipping include diabetes mellitus, obstructive sleep apnea, chronic renal failure, and secondary causes of hypertension (2–4). A recent report suggested that atherosclerosis-related dysfunction in the baroreceptor may be related to poor nocturnal dipping in patients with stroke (5). The “non-dipping” and “reverse dipping” patterns may result in a higher 24-h mean blood pressure level leading to greater pressure load and shear stress on vessels resulting in accelerated atherosclerosis (4, 6). There is also a link between resting heart rate and vascular disease (7–11). Similar to changes in nocturnal blood pressure, non-dipping in the heart rate during sleep is associated with an increased risk of heart disease and increased mortality (7–10).

White matter hyperdensities and small vessel disease (SVD) increase with age and are increasingly being recognized in patients with hypertension and other vascular risk factors. The presence of SVD increases the risk of stroke, CAD, cognitive dysfunction and dementia, and mortality (12). Several reports from Japan and South Korea have documented an increase in the incidence of SVD in hypertensive subjects non-dipping on prolonged ambulatory monitoring. A similar increase in SVD has recently been reported in community-dwelling elderly Japanese with blunted nocturnal heart rate dipping (11). The effects of nocturnal non-dipping in blood pressure and heart rate on the severity of acute stroke or rates of post-stroke recovery and prognosis have not been studied.

We have previously reported on the high incidence of SVD in multi-ethnic patients with acute stroke from Qatar. SVD was evident in 65% of subjects, with an even higher prevalence noted in patients with preexisting hypertension and diabetes (13). Ambulatory blood pressure monitoring (ABPM) was, however, not done in these patients and we did not determine the relationship between non-dipping patients and prognosis following acute stroke.

In this study, we report on the effects of nocturnal non-dipping of blood pressure and heart rate on the frequency of SVD in acute stroke patients. We studied the 24-h ambulatory blood pressure (ABPM) and heart rate in acute stroke patients in whom MRI scans were performed during admission. Our primary objective was to determine the frequency of non-dippers and quantify the severity of SVD in dippers vs. non-dippers. We also examined the association of SVD in non-dippers on the severity of symptoms at presentation and the 90-day prognosis.

## Methods

The study was approved by the Institutional Review Board at Hamad Medical Corporation (HMC-IRB, 16428/16). All acute stroke patients admitted to the Hamad General Hospital (HGH) from September 2018 to October 2020, were approached for the study. Following consent, the measurement of the ambulatory pressure was completed in most patient during the admission, usually during day 2–7 of the admission. The MRI was completed within 2–4 days of the admission. For the current study, we only included patients in whom ABPM and MRI were completed during the admission. The clinical data, laboratory investigations, and imaging information were collected prospectively and entered into the case record forms (CRFs).

### Study Population

The clinical information was collected on all consented patients admitted to the HGH including prehospital modified Rankin Scale (mRS), risk factors for stroke, mode of presentation, the severity of symptoms (as measured by the National Institute of Health Stroke Scale [NIHSS]), evaluation and investigations in the emergency department, complications in hospital, final diagnosis and prognosis at discharge and mRS following discharge at 90 days. The information was collected in a database. The details of the database have previously been described (13, 14).

### Risk Factor Assessment and Ambulatory Blood Pressure Monitoring

Cardiovascular risk factors were ascertained through direct examination and interviews conducted by trained clinical nurse specialists. Diabetes mellitus was defined by the current use of insulin or hypoglycemic agents or admission HbA1c more than 7.0%. Hypercholesterolemia was defined as a total serum cholesterol >5.2 mmolL, or the use of lipid-lowering medications. Body mass index was calculated using height and weight (kg/m2). Hospital admission systolic BP (SBP) and diastolic BP (DBP) were measured on the non-dominant arm in a lying position, using a sphygmomanometer calibrated against a reference mercury sphygmomanometer and with an arm cuff of appropriate size. BPs were recorded twice with a 5-min interval, and the average of the two recordings was used. Hypertension was defined as office SBP >140 mmHg or DBP >90 mmHg (mean of two readings), or prior antihypertensive medication use. We also included patients with no previous history of hypertension in whom the diagnosis was entertained when the blood pressure reading was consistently more than SEP >140 or DBP >90 during multiple readings during the admission.

Ambulatory blood pressure monitoring (ABPM) was performed with B-Pro (B-Pro^®^ HealthSTATS International, Singapore, Singapore) on the non-dominant arm. The methods of ambulatory BP monitoring with B-Pro have been compared to standard arm cuff devices with comparable results (15). The B-pro was attached to the wrist just proximal to the hand over the radial artery between 7 and 10 am. The patient's sleep onset and wake-up times were recorded. Ambulatory BP readings were automatically taken and recorded every 15 min during waking and sleeping hours for 24 h. The mean SBP and DBP were calculated for the 24-h period and separately for daytime (awake) and night-time (sleep) periods, defined by subjects' diary reports of actual asleep and awake times. Elevated ambulatory BPs were defined as follows; elevated 24-h ABP as mean 24-h SBP >130 mmHg and/or DBP >80 mmHg; elevated daytime ABP as mean daytime SBP >135 mmHg and/or DBP >85 mmHg; and elevated night-time ABP as mean night-time SBP >120 mmHg and/or DBP >70 mmHg (16). Nighttime dipping was calculated from ABPM measures, including night-to-day SBP ratio (mean night-time SBP/mean daytime SBP), and calculated and categorized them into two patterns: dipping and non-dipping pattern (17). A “non-dipping BP” is usually defined as a nocturnal BP fall of <10%.

The gold standard method of measuring the central aortic systolic pressure (CASP) is *via* direct measurement with an intra-aortic transducer. However, the method is invasive and several non-invasive methods are available that allow for comparable measurements (18). The B-Pro ABPM monitoring system can measure the CASP (systolic and mean) and the accuracy has been validated with other similar devices (15, 18). We had detailed CASP measurements available in 180 patients. To evaluate if an increase in CASP may contribute to SVD, the measurements were compared in patients with or without SVD as measured on MRIs.

The heart rate data was obtained from the B-pro monitors and was available for 180 of the patients. We used the methods as defined by Ogoyama et al. (9) for calculation of the day time, night time, and dipping status. In brief, the following formula was used for the calculation of night-time dipping (daytime heart rate—nighttime heart rate/daytime heart rate). A change of <10% was considered as non-dipping. The dipping percentage was defined as daytime heart rate—nighttime heart rate x 100/daytime heart rate (9).

### MRI Image Acquisition of the Brain and Interpretation

Brain images were obtained on a Siemens 3T MRI. The details of the methodology have been previously published in a report from Qatar in a similar population (13). Briefly, all patients had the following MRI sequences performed; diffusion-weighted imaging, apparent diffusion coefficient, axial T1, T2, fluid-attenuated inversion recovery, sagittal T1, coronal T2 susceptibility-weighted imaging, contrast-enhanced sagittal T1 three-dimensional magnetization prepared rapid acquisition gradient echo (MPRAGE) with axial and coronal reformats, and three-dimensional time of flight intracranial and post-contrast cervical MR angiography were obtained for each subject with a 3.0-T superconducting magnet (MAGNETOM Skyra, Siemens, Germany). For diagnosis of SVD, we included silent stroke (SS), periventricular hyperintensities (PVHIs), white matter hyperintensities (WMHIs), and cerebral microbleeds (CMBs) using the Fazekas scale (19) as discussed in detail by Pantoni et al. and Kullar et al. (20, 21). Silent stroke was defined as focal hyperintensities on T2-weighted images, 3 mm in size or larger. Cerebral infarction was defined as “silent” if there was no corresponding history of a stroke or TIA. A combination of T1, T2, and fluid-attenuated inversion recovery (FLAIR) scans was used to distinguish infarcts from dilated perivascular spaces. Lesions in the white matter also required corresponding prominent hypointensities on T1-weighted images, to distinguish them from other cerebral white matter abnormalities. Lacunes were defined as focal lesions between 3 and <15 mm seen on FLAIR, T1-weighted, and T2-weighted sequences. If lacunes were more than 15 mm, they were defined as subcortical infarcts, and if the cortical gray matter was affected, they were classified as cortical infarcts. A modification of previously published scales was used to describe the different types of hyperintense signal abnormalities surrounding the ventricles and in the deep white matter (22, 23). A similar methodology has been used in previous studies (2). PVHI was graded as 0 = absence, 1 = “caps” or pencil-thin lining, 2 = smooth “halo,” and 3 = irregular PVHI extending into the deep white matter. Separate deep WMHIs were rated as 0 = absence, 1 = punctate foci, 2 = beginning confluence of foci, and 3 = large confluent areas. For the current analysis, a score of 2 or more was considered significant for WMHIs and PVHIs. Cerebral microbleeds were defined as round-shaped homogenous foci of low signal intensity lesions <5 mm on SWI (24, 25). The foci located in the subarachnoid spaces or symmetrically in the globus pallidus were not included because these may be confused as vessel markings heavy mineral deposits, or calcifications. The locations of CMBs were classified as lobar, central gray matter (basal ganglia and thalamus), white matter (periventricular and white matter), and infra-tentorial area (brainstem and cerebellum) (26). The MR studies were performed under the direct supervision of the neuroradiologists in our team, who officially report the studies. The reviewer was unaware of the clinical diagnosis or the ABPM results at the time of the rating of the MRI.

### Statistical Methods

Descriptive results for all continuous variables were reported as mean ± standard deviation and numbers (percentage) for categorical variables. Median and Inter Quartile Range (IQR) were calculated for NIHSS score. Student *t*-tests for continuous variables and chi-square tests for categorical variables were performed between the groups (Nocturnal Dippers vs. Nocturnal Non-Dippers) and (SVD Absent vs. SVD present). Multivariable logistic regression analyses were used to evaluate the association of ABM measures with subclinical cerebrovascular disease. Multivariable models were adjusted for the factors associated with subclinical cerebrovascular disease at the *P* < 0.05 level in the univariable analyses of ABPM and brain MRI abnormalities patients. Odds ratios and 95% confidence interval were reported. ROC curve with c-statistics was presented to see the model accuracy for SVD. A *p* < 0.05 (two-tailed) was considered statistical significant level. SPSS 21·0 statistical package was used for the analysis.

## Results

There were 234 patients in whom ABPM was completed and MRI data was available. The 24-h pulse reading was available in 180 patients. In the remainder of subjects, the pulse recordings were not of sufficient quality to have an accurate determination of the daytime and nighttime measurements accurately and were therefore not utilized for further analysis.

The demographics, risk factors, current medications at the time of admission, clinical diagnosis, and the severity of stroke symptoms are shown in Table 1. Overall, there were 162 patients (69.2%) in whom nocturnal blood pressure dipping was not observed. There were no significant differences in the risk factors (including hypertension) between dippers and non-dippers. At admission 175 (74.8%) patients were known to have hypertension. There were 126 (77.8%) patients who were non-dippers in this group. Twenty-nine additional patients were diagnosed with hypertension during the hospitalization (total: 191 including 51 [26.7%] dippers and 140 [73.3%] non-dippers).

**Table 1:** Demographic features, stroke severity, outcome, and the presence of SVD in the patients with and without nocturnal dipping.

**Characteristic or investigations**	**Total** **(*n* = 234)**	**Nocturnal dippers** **(*n* = 72, 30.8%)**	**Nocturnal non-dippers** **(*n* = 162, 69.2%)**	***P* value**
Age, Mean, years	51.1 ± 9.9	50.531 ± 0.3	51.359 ± 0.8	0.56
Men (%)	205 (87.6)	64 (88.9)	141 (87.0)	0.69
Hypertension on admission	175 (74.8)	49 (68.1)	126 (77.8)	0.11
Diabetes on admission	97 (41.5)	31 (43.1)	66 (40.7)	0.74
Dyslipidemia on admission	96 (41.0)	36 (50.0)	60 (37.0)	0.06
CAD on admission	13 (5.6)	4 (5.6)	9 (5.6)	1.00
AF on admission	6 (2.6)	3 (4.2)	3 (1.9)	0.30
Active smoking	75 (32.1)	24 (33.3)	51 (31.5)	0.78
History of stroke	15 (6.4)	6 (8.3)	9 (5.6)	0.42
Obese (≥30.0 Kg/m2)	73 (31.2)	20 (27.8)	53 (32.7)	0.45
BMI (kg/m2)	28.4 ± 5.1	27.7 ± 3.9	28.9 ± 5.6	0.17
SBP (mm Hg)	156.0 ± 26.1	156.6 ± 24.9	157 ± 26.7	0.53
DBP (mm Hg)	92.9 ± 18.8	94.1 ± 18.3	92.4 ± 19.0	0.17
NIHSS on admission (Median, -IQR)	1 (0–4)	1 (0–4)	1 (0–4)	0.66
Stroke severity				
Mild stroke (NIHSS 0–4)	194 (82.9)	61 (84.7)	133 (82.1)	0.74
Moderate stroke (NIHSS 5–10)	30 (12.8)	9 (12.5)	21 (13.0)	
Severe stroke (NIHSS 11 or more)	10 (4.3)	2 (2.8)	8 (4.9)	
Final diagnosis				
Ischemic stroke	157 (67.1)	48 (66.7)	109 (67.3)	O.89
Transient ischemic attack	55 (23.5)	18 (25)	37 (22.8)	
Intracerebral hemorrhage	22 (9.4)	6 (8.3)	16 (9.9)	
TOAST classification				
Small vessel disease	93 (58.9)	63 (62.5)	30 (57.3)	0.07
Large vessel disease	23 (14.6)	2 (4.2)	21 (19.1)	
Cardio-embolic	26 (16.5)	8 (16.7)	18 (16.4)	
Stroke of determined origin	5 (3.2)	3 (6.3)	2 (1.8)	
Stroke of undetermined origin	11 (7.0)	5 (10.4)	6 (5.5)	
NIHSS at discharge (Median,- IQR)	1 (0–2)	1.8 ±2.6 0 (0–3)	1.9 ±2.9 1 (0–2)	0.71
Prognosis –at discharge –good (mRS 0–2)	182 (77.8)	56 (77.8)	126 (77.8)	1.00
Prognosis – At 90-days –good (mRS 0–2)	223 (95.3)	68 (94.4)	155 (95.7)	0.68
Average 24-h SBP at index event	142.7 ± 20.9	139.2 ± 19.3	144.3 ± 21.6	0.09
Average 24 h DBP at Index event	90.6 ± 15.1	88.8 ± 14.9	91.4 ± 15.2	0.23
Average daytime SBP at index event	145.9 ± 21.4	146.0 ± 20.3	145.7 ± 22	0.96
Average daytime DBP at index event	145.9 ± 21.4	92.6 ± 16.4	92.6 ± 15.5	0.99
Average nighttime SBP at index event	92.6 ± 15.7	127.2 ± 18.7	141.4 ± 22.4	0.001
Average nighttime DBP at index event	137.0 ± 22.3	80.9 ± 14.1	89.4 ± 15.3	0.001
Mean dipping % at index event		13.3 ± 2.6	4.5 ± 2.9	0.001
MRI findings at index event				
Small vessel disease present	121 (51.7)	29 (40.3)	92 (56.8)	0.02
New stroke on imaging	170 (72.6)	54 (75.0)	116 (71.6)	0.59
Silent infracts present	85 (36.3)	19 (26.4)	66 (40.7)	0.035
White matter disease (periventricular) present	183 (78.2)	51 (70.8)	132 (81.5)	0.069
White matter disease (brainstem) present	38 (16.2)	6 (8.3)	32 (19.8)	0.029
Cerebral microbleed present	50 (21.4)	12 (16.7)	38 (23.5)	0.24
Severity of CMB at index event				
NO CMB	184 (78.6)	60 (83.3)	124 (76.5)	0.50
CMB 1–4	34 (14.5)	8 (11.1)	26 (16.0)	
CMB 5 or more	16 (6.8)	4 (5.6)	12 (7.4)	

*CAD, Coronary Artery Disease; AF, Atrial Fibrillation; BMI, body mass index; SBP, systolic blood pressure; DBP, diastolic blood pressure; DM, diabetes mellitus; HbA1c, hemoglobin A1c; HDL, high-density lipoprotein; LDL, low-density lipoprotein; NIHSS, the National Institutes of Health Stroke Scale; IQR, Inter Quartile Range; mRS modified small vessel disease; SVD small vessel disease; CMB cerebral microbleed*.

Small vessel disease was evident in 121 of 234 (51.7%) patients as shown in Table 2. Patients with SVD were significantly older with no sex dominance. Significantly more patients with SVD had a history of hypertension and previous stroke and CAD. Compared to nocturnal dippers, SVD was significantly higher in non-dippers (56.8 vs. 40.3% *p* = 0.02). The differences in the severity of SVD changes were most evident in the brainstem region (19.8 vs. 8.3% *p* = 0.02). Periventricular changes were marginally higher in non-dippers (81.5 vs. 70.8% *p* = 0.069). Silent infarctions were significantly more frequently seen in non-dippers compared to dippers (40.7% vs. 26.4% *P* = 0.03). CMBs were also marginally increased in non-dippers but did not reach significance. In all, CMBs were evident in 21.4% of patients (non-dippers 23.5% and dippers 16.7% *P* = 0.24). Patients with nocturnal pulse dipping were also more likely to have significantly fewer silent strokes and small vessel disease as shown in Table 3. CMBs appeared to have no relationship with pulse dipping.

**Table 2:** Demographics, risk factors, clinical presentation, severity of symptoms at presentation, short-term prognosis in patients with and without small vessel disease (SVD).

**Characteristic or investigations**	**Total** **(*n* = 234)**	**SVD ABSENT** **(*n* = 113, 48.3%)**	**SVD PRESENT** **(*n* = 121, 51.7 %)**	***P* value**
Age, Mean, years	51.1 ± 9.9	48.6 ± 9.4	53.4 ± 9.9	<0.001
Men (%)	205 (87.6)	98 (86.7)	107 (88.4)	0.69
Dipping status				
Normal dippers	72 (30.8)	43 (38.1)	29 (24.0)	0.02
Non-dippers	162 (69.2)	70 (61.9)	92 (76.0)	
Hypertension on admission	175 (74.8)	71 (62.8)	104 (86.0)	<0.001
Diabetes on admission	97 (41.5)	42 (37.2)	55 (45.5)	0.19
Dyslipidemia on admission	96 (41.0)	46 (40.7)	50 (41.3)	0.92
Coronary artery disease	13 (5.6)	1 (0.9)	12 (9.9)	0.003
Atrial fibrillation on admission	6 (2.6)	4 (3.5)	2 (1.7)	0.36
Active smoking	75 (32.1)	31 (27.4)	44 (36.4)	0.14
History of stroke	15 (6.4)	3 (2.7)	12 (9.9)	0.023
BMI (kg/m2)	28.4 ± 5.1	27.9 ± 4.6	28.8 ± 5.6	0.20
HbA1c %	6.8 ± 2.1	6.6 ± 1.8	7.1 ± 2.3	0.04
Total cholesterol	4.6 ± 1.3	4.6 ± 1.3	4.5 ± 1.3	0.94
Serum triglyceride	1.7 ± 0.9	1.8 ± 1.0	1.7 ± 0.8	0.53
HDL cholesterol	1.1 ± 0.4	1.1 ± 0.4	1.0 ± 0.4	0.22
LDL cholesterol	2.9 ± 0.9	2.9 ± 0.9	2.8 ± 0.9	0.67
Admission NIHSS (median IQR)	1 (0–4)	1 (0–3)	2 (0–4)	0.002
Stroke severity				
Mild stroke (NIHSS 0–4)	194 (82.9)	102 (90.3)	92 (76.0)	0.013
Moderate stroke (NIHSS 5–10)	30 (12.8)	9 (8.0)	21 (17.4)	
Severe stroke (NIHSS 11 or more)	10 (4.3)	2 (1.8)	8 (6.6)	
Final diagnosis				
Ischemic stroke	157 (67.1)	73 (64.6)	84 (69.4)	0.16
Transient ischemic attack	55 (23.5)	32 (28.3)	23 (19.0)	
Intracerebral hemorrhage	22 (9.4)	8 (7.1)	14 (11.6)	
TOAST classification				
Small vessel disease	93 (58.9)	37 (50.7)	56 (65.9)	0.17
Large vessel disease	23 (14.6)	10 (13.7)	13 (15.3)	
Cardio-embolic	26 (16.5)	15 (20.5)	11 (12.9)	
Stroke of determined origin	5 (3.2)	3 (4.1)	2 (2.4)	
Stroke of undetermined origin	11 (7.0)	8 (11.0)	3 (3.5)	
NIHSS at discharge (median IQR)	1 (0–2)	0 (0–2)	1 (0–3)	0.03
Prognosis at discharge-good (mRS 0–2)	182 (77.8)	93 (82.3)	89 (73.6)	0.11
Prognosis—at 90-days—good (mRS 0–2)	223 (95.3)	110 (97.3)	113 (93.4)	0.15
Average 24 h SBP at index event	142.7 ± 20.9	138.9 ± 18.9	146.3 ± 22.2	0.007
Average 24 h DBP at Index event	90.6 ± 15.1	89.9 ± 14.1	91.1 ± 16.0	0.56
Average daytime SBP at Index event	145.9 ± 21.4	142.3 ± 19.8	149.2 ± 22.5	0.014
Average daytime DBP at index event	145.9 ± 21.4	91.9 ± 15.3	93.2 ± 16.1	0.53
Average nighttime SBP at index event	92.6 ± 15.7	132.2 ± 19.0	141.5 ± 24.1	0.001
Average nighttime DBP at index event	137.0 ± 22.3	85.6 ± 14.1	88.0 ± 16.5	0.23
Mean dipping % at index event	7.2 ± 4.9	7.9 ± 5.0	6.6 ± 4.9	0.051

*CAD, Coronary Artery Disease; AF, Atrial Fibrillation; SBP, systolic blood pressure; BMI, body mass index; DBP, diastolic blood pressure; DM, diabetes mellitus; HbA1c, hemoglobin A1c; SBP, systolic blood pressure; HDL, high-density lipoprotein; LDL, low-density lipoprotein; NIHSS, the National Institutes of Health Stroke Scale; IQR, Inter Quartile Range; mRS modified small vessel disease; SVD small vessel disease; CMB, cerebral microbleed*.

**Table 3:** Demographics, clinical features, and imaging characteristics of the patients with and without nocturnal pulse dipping.

**Characteristic or investigations**	**Total** **(*n* = 180)**	**Nocturnal pulse dippers (*n* = 59, 32.8%)**	**Nocturnal pulse non-dippers (*n* = 121, 67.2%)**	***P* value**
Age, Mean, years	51.3 ± 9.9	51.2 ± 10.2	51.8 ± 9.8	0.89
Male (%)	157 (87.2)	51 (86.4)	106 (87.6)	0.83
Hypertension on admission	137 (76.1)	41 (69.5)	96 (79.3)	0.15
Diabetes on admission	75 (41.7)	23 (39.0)	52 (43.0)	0.61
Dyslipidemia on admission	73 (40.6)	25 (42.4)	48 (39.7)	0.73
Coronary artery disease	8 (4.4)	0	8 (6.6)	0.04
Atrial fibrillation on admission	5 (2.8)	3 (5.1)	2 (1.7)	0.19
Active smoking	54 (30.0)	16 (27.1)	38 (31.4)	0.56
History of stroke	9 (5.0)	2 (3.4)	7 (5.8)	0.49
Obese (≥30.0 Kg/m2)	61 (33.9)	16 (27.1)	45 (37.2)	0.18
BMI (kg/m2)	28.7 ± 5.5	28.4 ± 5.9	28.8 ± 5.3	0.66
NIHSS on admission (median, IQR)	1 (0–4)	1 (0–4)	1 (0–3)	0.87
NIHSS at discharge (median, IQR)	1 (0–2)	1 (0–2)	1 (0–2)	0.47
Stroke severity on admission				
Mild stroke (NIHSS 0–4)	152 (84.4)	52 (88.1)	100 (82.6)	0.32
Moderate stroke (NIHSS 5–10)	21 (11.7)	4 (6.8)	17 (14.0)	
Severe stroke (NIHSS 11 or more)	7 (3.9)	4 (5.1)	3 (3.3)	
Final diagnosis				
Ischemic stroke	120 (66.7)	44 (74.6)	76 (62.8)	0.19
Transient ischemic attack	42 (23.3)	12 (20.3)	30 (24.8)	
Intracerebral hemorrhage	18 (10.0)	3 (5.1)	15 (12.4)	
TOAST classification				
Small vessel disease	73 (60.3)	29 (65.9)	44 (57.1)	0.19
Large vessel disease	15 (12.4)	4 (9.1)	11 (14.3)	
Cardio-embolic	20 (16.5)	4 (9.1)	16 (20.8)	
Stroke of determined origin	4 (3.3)	3 (6.8)	1 (1.3)	
Stroke of undetermined origin	9 (7.4)	4 (9.1)	5 (6.5)	
Prognosis—at discharge				
Good (mRS 0–2)	142 (78.9)	46 (78.0)	96 (79.3)	0.83
Poor (mRS 3–6)	38 (21.1)	13 (22.0)	25 (20.7)	
Prognosis—at 90-days				
Good (mRS 0–2)	171 (95.0)	56 (94.9)	115 (95.0)	0.97
Poor (mRS 3–6)	9 (5.0)	3 (5.1)	6 (5.0)	
Small vessel disease present	94 (52.2)	24 (40.7)	70 (57.9)	0.03
Silent infracts present	69 (38.3)	15 (25.4)	54 (44.6)	0.013
White matter disease present	140 (77.8)	38 (64.4)	102 (84.3)	0.003
Cerebral microbleed present	43 (23.9)	13 (22.0)	30 (24.8)	0.68

*BMI, body mass index; HbA1c, hemoglobin A1c; HDL, high-density lipoprotein; LDL, low-density lipoprotein; NIHSS, National Institutes of Health Stroke Scale; TOAST, Trial of ORG 10172 in Acute Stroke Treatment) classification; bpm, Beats Per Minute*.

Table 4 shows multivariate regression analysis on 234 subjects available for all the variables. Adjusting for the effect of clinical variables identified by univariable analyses age, sex, history of hypertension, dipping status, the severity of stroke as measured with NIHSS, and prior history of stroke or CAD, hypertension on admission (adj OR: 2.87, 95% C.I.: 1.45–5.67, *p* = 0.002), non-dippers (adj OR: 1.95, 95% C.I.: 1.04–3.65, *p* = 0.04), and NIHSS on admission (adj OR: 1.15, 95% C.I.: 1.04–1.28, *p* = 0.007) were found significantly associated with SVD.

**Table 4:** Multivariate analysis of the variables associated with SVD.

**VARIABLE**	**ODDS RATIO**	**95% CI**		**P Value**
		**Lower**	**Upper**	
Age	0.004	1.02	1.07	1.08
Sex	1.25	0.53	2.97	0.61
BP non-dipper	1.95	1.04	3.65	0.04
NIHSS on admission	1.15	1.04	1.28	0.007
Prior stroke history	3.43	0.83	14.2	0.09
Hypertensive on admission	2.87	1.45	5.67	0.002
Coronary artery disease	6.99	0.84	58.17	0.07

*Results are adjusted for the factors including age, sex, history of hypertension, dipping status, admission NIHSS, prior history of stroke, coronary artery disease, and non-dippers and correlated its association with small vessel disease (SVD)*.

**Figure 1:**
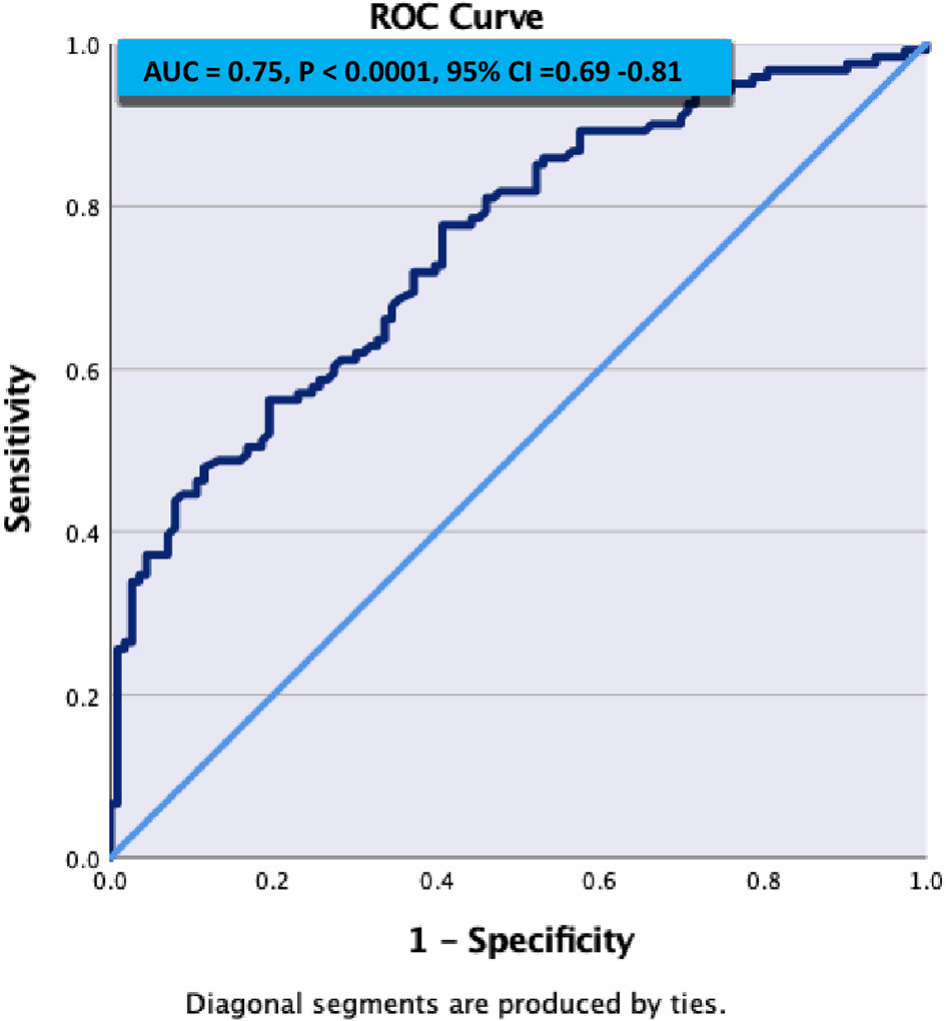
ROC curve to predictive accuracy of SVD from the model.

Figure 1 demonstrates C- statistics in the form of receiver operative Curve (ROC) suggested that the model is able to discriminate 75% accurately between SVD and Non SVD. When similar multivariate regression analysis was performed including pulse non-dipper varaibale having only 180 subjects, only NIHSS on admission (adj OR: 1.24, 95% C.I.: 1.08–1.44, *p* = 0.003) and hypertensive on admission (adj OR: 3.14, 95% C.I.: 1.35–7.28, *p* = 0.01) were found significantly associated with SVD (Table 5).

**Table 5:** Multivariate analysis after adding pulse non-dipping status to see the association with SVD.

**VARIABLE**	**ODDS RATIO**	**95% CI**		***P* value**
		**Lower**	**Upper**	
Age	1.03	0.99	1.07	0.07
Sex	1.29	0.47	3.56	0.63
BP non-dipper	1.97	0.78	4.98	0.15
Pulse non-dipper	1.31	0.58	2.94	0.51
NIHSS on admission	1.24	1.08	1.44	0.003
Prior stroke history	6.31	0.72	55.32	0.09
Hypertensive on admission	3.14	1.35	7.28	0.008

*Results are adjusted for the factors including age, sex, history of hypertension, dipping status, admission NIHSS, prior history of stroke, coronary artery disease, and non-dippers and correlated its association with small vessel disease (SVD)*.

## Discussion

The prevalence of non-dipping blood pressure is ~30% in normotensive individuals and higher in hypertensive individuals (27, 28) and has been associated with a higher risk of recurrent stroke and poor outcome (29, 30). In our study, 69% of patients were nocturnal non-dippers (74.3% non-dippers in hypertensive patients) who suffered an acute stroke. This is somewhat lower than the non-dipper rates of 89.5% in patients with acute stroke reported by Rejmar et al. (31). The significantly younger age at presentation of stroke symptoms in our patients may account for the lower rates. The presence of non-dipping was associated with a significantly higher number of patients in whom the MRI showed SVD and silent infarctions. Also, patients in whom nocturnal non-dipping was evident and who had SVD on MRI, were more likely to have a severe stroke. Whereas our research does not have prolonged follow-up, other studies have shown that patients with nocturnal non-dipping patterns are at a higher risk of stroke and CAD (2–5). Finally, nocturnal non-dipping of the heart rate was also associated with significantly higher rates of silent strokes and SVD.

Small vessel disease is an important risk factor for stroke, cognitive impairment, gait disturbance, and early mortality (12). The underlying pathogenic mechanisms include endothelial cell damage, blood-brain barrier dysfunction, white matter rarefaction, inflammation, and ischemia. Increasing microvascular changes resulting in chronic hypoperfusion and ischemia and dysregulation of glial cells, such as oligodendrocytes and astrocytes, are likely important initiators of SVD (32). Hypertension, especially systolic hypertension, is an important factor associated with the progression of SVD (33) and preliminary studies suggest that effective control of hypertension may slow the progression of SVD (33, 34) although recent results from the SPRINT-MIND trial did not show slowing of SVD progression in the arm “aggressive” blood pressures management (35). In our study, severe stroke at presentation was present in patients with SVD. The presence of SVD has also previously been reported with slower recovery following acute stroke (20).

Blood pressure normally decreases during the night and reaches its trough during the deep stages of sleep (nocturnal dipping) and begins to rise in the morning as the person awakens (morning surge) (2). The reversal of nighttime dipping is seen most frequently in patients with hypertension and is considered to be an important marker for accelerated atherosclerosis, CAD, and stroke (6). Nocturnal non-dipping is also associated with an increased risk of silent asymptomatic SVD in observational brain imaging studies. In a follow-up study from Japan in 519 elderly subjects with silent strokes on MRI, silent and symptomatic ischemic stroke and cerebral hemorrhage were most frequently seen in patients with extreme dipping and reverse dipping (36). In a recent meta-analysis of 12 studies evaluating the relationship of blood pressure and presence of silent stroke and SVD, the lesions were twice more likely in nocturnal non-dippers and reverse dippers when compared to subjects with normal dipping patterns (4). Interestingly and similar to our observations, non-dipping was not associated with an increased risk of CMBs (4). The frequency of SVD in most reported studies was on 1.5T MRIs and may be less sensitive to picking up SVD. All patients in our series had the studies performed on 3T magnets. The pathophysiological mechanism of why SVD is more common with non-dippers or reverse dippers is more common is not fully understood. The higher mean 24-h pressure level in such patients may have a higher overall load and stress to the vessels and the resulting damage to the endothelial cells may accelerate atherosclerosis and small-vessel occlusion (37).

A recent meta-analysis reported on short and long-term prognosis following acute stroke and the use of ABPM. Higher blood pressures during the day or night were a marker of poor outcome whereas nocturnal dipping was associated with a good prognosis (29). The presence of non-dipping was also found independently to be associated with an increased risk for silent strokes and symptomatic lacunar strokes during follow-up in a study from Japan (30). In our study, non-dippers, who also had SVD on MRI, presented with more severe stroke symptoms. Nocturnal non-dipping and reverse dipping have previously been shown to be associated with more severe acute stroke symptoms when compared to dippers in 24-h ABPM studies (38).

Surprisingly, nocturnal heart rate non-dipping has received limited attention (40). It is associated with an increase in cardiovascular disease (39) and, increased overall mortality (39). Similar to our data, there are rare reports of an increase in SVD in patients with nocturnal non-dipping of heart rates (10, 40, 41). The mechanisms for accelerated atherosclerosis with pulse non-dipping is likely similar to that seen in blood pressure non-dipping (11).

Our study has limitations. The number of patients with ABPM was small and the 24-h heart rate measurements were available in an even smaller number of patients. A larger series of patients may have allowed for better characterization of trends noted in some additional variables. The patients in our series were young, and the majority had acute lacunar strokes with mild symptoms. This may not be representative of stroke in Western countries and the results cannot be generalizable. Other reports of ABPM within 24–48 h of an acute stroke have also shown the importance of nocturnal non-dipping and prognosis (38, 41). Another limitation of the study is that the MRI and the ABPM were done during the same admission. Because of the limited number of patients, we were unable to evaluate other important confounders in the multivariate analysis. Finally, we have a limited follow-up on our patients. This may not allow for the prognostic significance of our observations.

In summary, we present the 24-h ABPM, heart rate, and MRI findings in a series of patients with acute stroke. Our study shows that hypertension and nocturnal non-dipping of blood pressure and heart rate is associated with an increase in silent stroke and SVD in patients presenting with an acute stroke. We also show that patients with pre-existing silent SVD present with more severe symptoms. The lack of long-term follow-up in our study is a limitation but several previous studies suggest that non-dipping may be associated with slower recovery and a higher risk of recurrent strokes (37). There is very little evidence on the best means to slow the progression of vascular disease in non-dippers. Indirect studies suggest that nighttime use of blood pressure medications, by restoring blood pressure, may slow down the risk of CAD (42, 43) although there is still controversy whether this may be effective in preventing CAD (44).

## Data Availability Statement

The original contributions presented in the study are included in the article/supplementary material, further inquiries can be directed to the corresponding author/s.

## Ethics Statement

The studies involving human participants were reviewed and approved by IRB of Hamad Medical Corporation. The patients/participants provided their written informed consent to participate in this study.

## Author Contributions

NA and AS: concept, design, and draft. NA, BB, DM, SJ, RF, and MA: acquisition, analysis, interpretation of data, technical, and administrative support. SK, SA-J, and AS: critical review. RS and NA: statistical analysis. All authors contributed equally to the manuscript.

## Funding

The authors received funding for this study from the Hamad Medical Corporation to cover manpower and from Weil Cornell College of Medicine-Qatar for procedures and publications.

## Conflict of Interest

The authors declare that the research was conducted in the absence of any commercial or financial relationships that could be construed as a potential conflict of interest.

## Publisher's Note

All claims expressed in this article are solely those of the authors and do not necessarily represent those of their affiliated organizations, or those of the publisher, the editors and the reviewers. Any product that may be evaluated in this article, or claim that may be made by its manufacturer, is not guaranteed or endorsed by the publisher.
